# Enhancement of the Antibacterial Activity of Natural Rubber Latex Foam by Blending It with Chitin

**DOI:** 10.3390/ma13051039

**Published:** 2020-02-26

**Authors:** Nanxi Zhang, Hui Cao

**Affiliations:** Beijing Key Laboratory of Bioprocess, College of Life Science and Technology, Beijing University of Chemical Technology, Beijing 100029, China; nancyzhang512@outlook.com

**Keywords:** natural rubber latex foam, chitin, antibacterial activity

## Abstract

To enhance the antibacterial activity of natural rubber latex foam (NRLF), chitin was added during the foaming process in amounts of 1–5 phr (per hundred rubber) to prepare an environmentally friendly antibacterial NRLF composite. In this research, NRLF was synthesized by the Dunlop method. The swelling, density, hardness, tensile strength, elongation at break, compressive strength and antibacterial activity of the NRLFs were characterized. FTIR and microscopy were used to evaluate the chemical composition and microstructure of the NRLFs. The mechanical properties and antibacterial activity of the NRLF composites were tested and compared with those of pure NRLF. The antibacterial activity was observed by the inhibition zone against *E. coli*. NRLF composite samples were embedded in a medium before solidification. The experimental results of the inhibition zone indicated that with increasing chitin content, the antibacterial activity of the NRLF composites increased. When the chitin content reached 5 phr, the NRLF composite formed a large and clear inhibition zone in the culture dish. Moreover, the NRLF–5 phr chitin composite improved the antibacterial activity to 281.3% of that of pure NRLF against *E. coli*.

## 1. Introduction

Natural rubber latex (NRL), the viscous liquid from *Hevea brasilensis*, is the main source of commercial natural rubber. Fresh NRL comprises an aqueous colloid with 44–70 wt% water. Generally, latex is processed into high ammonia natural rubber latex (HA-NRL) for convenience of preservation and transportation [1]. The main component of natural rubber is poly(cis-1,4-isoprene). In addition to water and rubber hydrocarbons, natural rubber latex also includes proteins, lipids and other substances [2,3].

Natural rubber latex products can be divided into four categories: impregnated products, molded products, extruded products and foam products. Compared with the former three, natural rubber latex foam (NRLF) is a porous material with a low density [4,5]. NRLF has the characteristics of elastic resilience, absorbency, providing sound insulation, being shockproof and providing ventilation [6,7,8,9,10,11,12]. At present, the commercial products made from NRLF mainly include mattresses and pillows. 

NRLF, widely used as bedding, is one of the economic mainstays of Southeast Asian countries [13]. However, NRLF is easily attacked by ultraviolet light due to its unsaturated carbon–carbon bonds [14]. NRLF bedding is not suitable for long-term exposure to sunlight, and slow bacterial growth might occur on the cover [15,16,17]. Therefore, it is necessary to improve the antibacterial activity of NRLF and to choose appropriate antibacterial agents to reduce the cost. 

**Figure 1:**
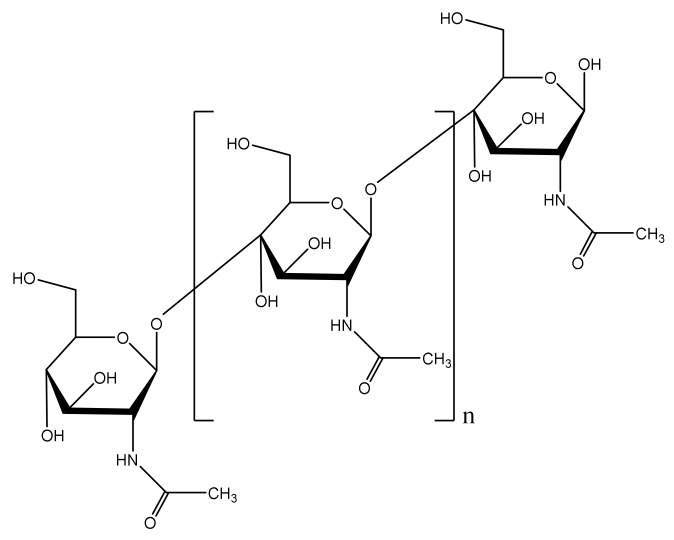
Chemical structural formula of chitin.

Chitin [(C_8_H_13_O_5_N)n], which widely exists in the shells of crustaceans such as shrimps and crabs, is a renewable, low-cost, antibacterial and well-sourced resource [18,19,20,21,22]. The chemical structure of chitin is shown in Figure 1. Chitin is a cationic natural polymer due to its amide groups. The positively charged polymer neutralizes the negatively charged functional groups on the surface of bacteria. Once the cell wall is damaged, the cell osmotic pressure would be destroyed and finally the bacteria would die [23,24]. Therefore, chitin could be considered an environmentally friendly antibacterial agent [25,26,27,28,29].

Most of the research on NRLF composites involves filler loading [30,31,32,33]. The focus is on exploring the mechanical properties of composite foams and emphasizing the reuse of natural fibers. Currently, the research on antibacterial NRLF mainly focuses on zinc oxide [34] and silver particles [35,36,37,38,39]. Khemara Mama [40] demonstrated that NRLF treated with silver nanoparticles of only 0.2 per hundred rubber (phr) had improved antibacterial ability by 43.8% against *E. coli* and 25% against *S. aureus* compared to NRLF that was not treated with silver nanoparticles. Few reports have been published on the utilization of natural renewable materials to prepare antibacterial NRLF composites.

Compared with zinc oxide and silver particles, chitin has the advantages of renewability, biodegradability and biocompatibility. Furthermore, the preparation of silver antimicrobials is quite complex due to the synthesis of nanoparticles [39] while blending with chitin is more simple and convenient. The poor solubility of chitin provides the NRLF composite with more durability within its service life. Therefore, chitin–NRLF composites can not only enhance the antibacterial activity of foam, but are also inexpensive and environmentally friendly [19].

In this research, 0, 1, 2, 3, 4 and 5 phr chitin-loaded NRLFs were prepared using the Dunlop method. Morphology, swelling, density, chemical composition, hardness, tensile strength, elongation at break and compressive strength are characterized to verify the antibacterial activity vs. mechanical properties of chitin–NRLF composites. This natural antibacterial composite foam has considerable prospects in commercial applications of natural rubber, which is consistent with the concept of sustainability and the goal of a green economy. 

## 2. Materials and Methods 

### 2.1. Materials 

The Dunlop method was used to prepare the NRLFs. High ammonia natural rubber latex (HA-NRL) was supplied by Yunnan Natural Rubber Industry Group Jingyang Co., Ltd (Yunnan, China). The chemicals (potassium hydroxide, 2-mercaptobenzimidazole, 2-mercaptobenzothiazole, sulfur, potassium oleate, ammonium sulfate, zinc oxide, sodium fluorosilicone), Luria-Bertani solid medium (deionized water, tryptone, sodium chloride, yeast extract, agar) and chitin were supplied by Sinopharm Chemical Reagent Co., Ltd. (China). *E. coli* BL21 were supplied by Qincheng Biotechnology Co., Ltd. (Shanghai, China). The names, concentrations and proportions of the chemicals used in the synthesis experiment are shown in Table 1.

### 2.2. Sample Preparation

The Dunlop method [41,42] involves prevulcanization, foaming, gelation, vulcanization and demolding. First, potassium hydroxide, 2-mercaptobenzimidazole, 2-mercaptobenzothiazole and sulfur were added into the HA-NRL. Prevulcanization of the NRL was carried out by stirring at 30 °C for 48 h at 50 rpm. Second, potassium oleate, ammonium sulfate and chitin were blended with the prevulcanized latex (PV-NRL). The PV-NRL was stirred for 8 min at 200 rpm to form a foam, and this process continued until the volume remained the same. Third, zinc oxide was added while the whisking machine was adjusted to 100 rpm. After 10 min, the sodium silicofluonate was slowly added, and the foam was stirred to the gelling point at 70 rpm. Once the gelling point was reached, the foam was injected into the mold and solidified at 90 °C for 30 min. Finally, the NRLF was vulcanized at 100 °C for 2 h. After demolding, washing and drying, the NRLF was obtained. The NRLFs loaded with chitin at 0, 1, 2, 3, 4 and 5 phr were prepared using the above procedure. Five samples were prepared for each condition.

### 2.3. Methods 

#### 2.3.1. Morphology

The microstructure and morphology of the NRLFs were observed with optical microscopy (Leica DM500, Heidelberg, German). The magnification (objective × eyepiece) was 40 ×. The samples were sliced for light transmission and observation. From the resulting graphs, the size of the micropores and the dispersion of the chitin particles were evaluated.

#### 2.3.2. Swelling

The NRLFs were made into 25 mm × 25 mm × 25 mm samples. The dried samples were weighed and then hermetically immersed in deionized water for 72 h. The surfaces of the samples were wiped with kitchen paper before the swollen samples were weighed. The testing temperature was 25 °C. The swelling is indicated as S (%).

S = (mass of swollen sample−mass of initial sample)/mass of initial sample(1)

#### 2.3.3. Density

The dry NRLFs were made into 50 mm × 50 mm × 25 mm (length × width × height) samples for measurement. The testing temperature was 25 °C. Five samples were tested under each loading condition, and the density is indicated as ρ (kg/m^3^).

#### 2.3.4. Chemical Composition

The chemical composition was characterized by FTIR (Nicolet iS50, Madison, WI, America). The transmittance of the samples to infrared light was measured. The chemical composition of the NRLFs with chitin loading (0, 1, 2, 3, 4 and 5 phr) was investigated in a mid-infrared region (500–4000 cm^−1^).

#### 2.3.5. Hardness

The hardness was tested according to the Shore C standard. A Shore C hardness tester (LX-C, Jiangdu, China) was used to measure the hardness of the soft foam materials. The dry NRLFs were made into 10 mm × 10 mm × 6 mm (length × width × height) samples for measurement. The testing temperature was 25 °C. Five samples were tested under each loading condition.

#### 2.3.6. Tensile Strength and Elongation at Break

Tensile strength and elongation at break were tested according to the Chinese National Standard GB/T 6344-2008. The NRLFs were made into 13 mm × 152 mm dumbbell-shaped samples with a gauge length of 50 mm. The speed of the universal material testing machine (XWW-20A, Beijing, China) used herein was 500 mm/min. The testing temperature was 25 °C. Five samples were tested under each loading condition.

#### 2.3.7. Compressive Strength

The NRLFs were made into 50 mm × 50 mm × 25 mm (length × width × height) samples. The compression ratio was 50%. The speed of the universal material testing machine (XWW-20A, Beijing, China) was 50 mm/min. The testing temperature was 25 °C. Five samples were tested under each loading condition.

#### 2.3.8. Antibacterial Activity

The antibacterial activity was characterized by the inhibition zone against *E. coli*. The NRLFs were made into cylindrical samples with a diameter of 8 mm and a height of 2 mm. The LB solid medium was poured into the culture dishes at 60 °C, and the samples were embedded in the medium. After the culture medium was completely solidified, bacterial liquid was added. All the dishes were incubated at 37 °C for 12 h.

## 3. Results

### 3.1. Morphology

**Figure 2:**
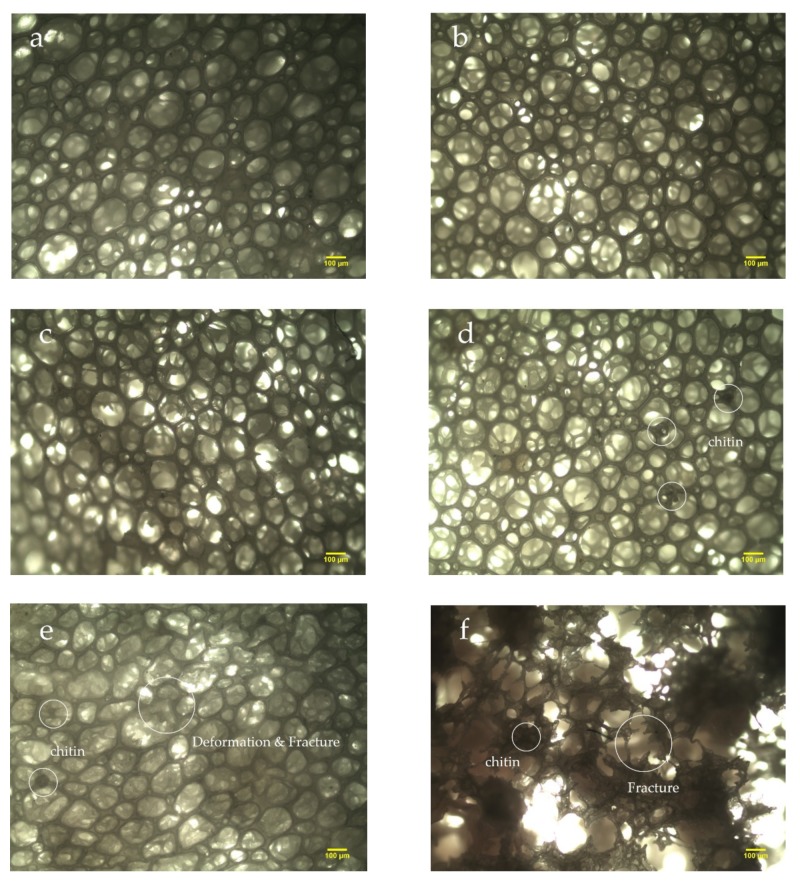
Micrographs of chitin–NRLF composites. (**a**) pure NRLF; (**b**) NRLF–1 phr chitin; (**c**) NRLF–2 phr chitin; (**d**) NRLF–3 phr chitin; (**e**) NRLF–4 phr chitin; (**f**) NRLF–5 phr chitin.

Figure 2 shows the micrographs of the NRLFs in different compositions. The microstructure, shape of the cells and dispersion of the chitin can be observed. Figure 2a–f shows the NRLFs loaded with 0–5 phr chitin. From Figure 2a–c, it is obvious that the shape of the cells changes little with a low chitin content. When the loading is 3 phr, the chitin begins to agglomerate on the walls. At the same time, the walls attached to the chitin would gain traction on the surrounding foam. Due to the influence of the chitin, the cells are no longer circular in shape as the loading increases.

The deformation and fracture of the cells were detected, as shown in Figure 2e. When the loading reaches 4 phr, the rubber walls fracture due to the over-expansion of the bubbles and the obstacles provided by the chitin. In Figure 2f, the number of cell cracks and the pore size increase. The broken foam wall adheres to the chitin. The pores are not stable enough, and chitin becomes aggregated, so the rubber begins to break under an uneven force. Macroscopically, small particles can be seen on the surface of the NRLF. The pores become large and weak with high filler contents.

**Figure 3:**
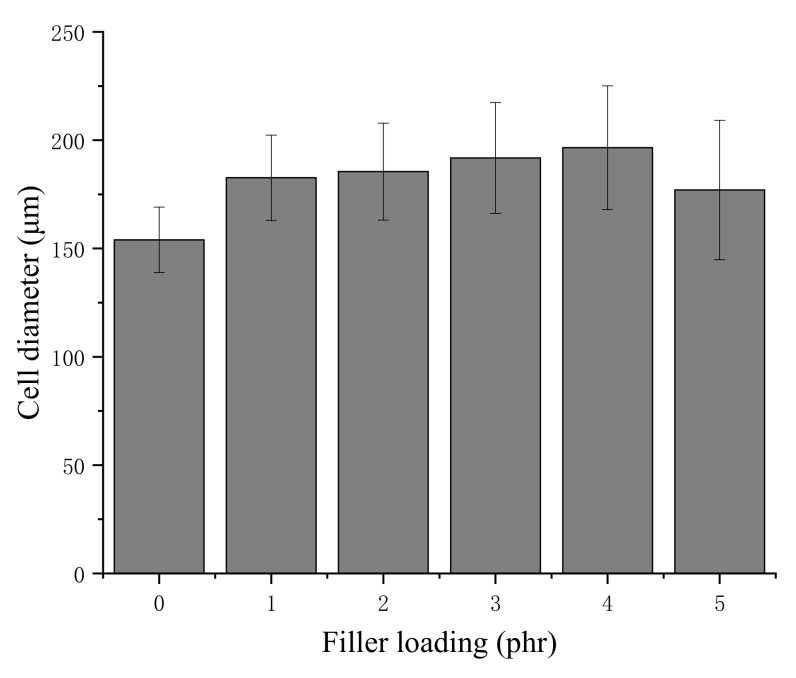
Cell diameters of chitin–NRLF composites.

The cell diameters of the NRLFs are shown in Figure 3. The relationship between pore size and filler loading can be seen in the diagram. First, as the loading of chitin increases, the pore size of the NRLF increases gradually. The large surface tension of the foam around the chitin results in the bursting and merging of the cells. However, the cell diameter in the NRLF–5 phr chitin case decreases. Under this loading, many bubbles become large enough to burst, and the remaining cells are weak and easily deformed. Therefore, only small cells could be measured completely. Second, with the increase in chitin loading, the standard deviation of the cell diameters increases as well. This indicates that the random dispersion of the chitin in NRLF leads to an uneven pore size. Compared with the cells in the NRLFs with a low chitin load, the difference among the cells in the NRLFs with a high chitin load is too large to be accurately estimated.

### 3.2. Swelling

**Figure 4:**
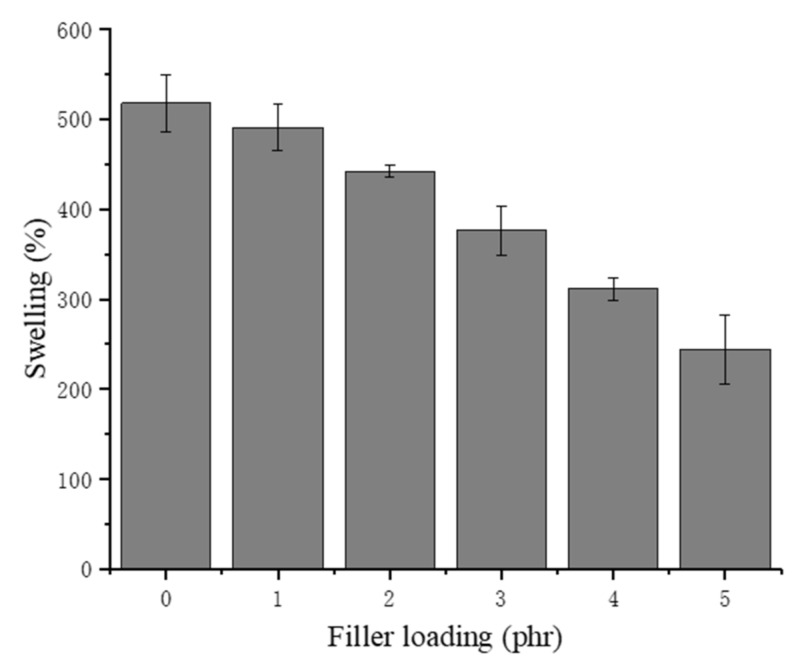
Swelling of chitin–NRLF.

The swelling ability could directly explain the porous structure of the NRLFs according to Equation (1). The NRLF composites were immersed in deionized water for 72 h, and the mass of the NRLF composites was measured. As shown in Figure 4, with increasing chitin content, the swelling percentage decreases. The reason might be the collapse and adhesion of the rubber foam when the loading increases. For NRLFs with a higher loading, the number of pores decreases sharply even though the cells merge and expand. Therefore, the low porosity of the NRLF composites diminishes the absorption capacity.

### 3.3. Density

**Figure 5:**
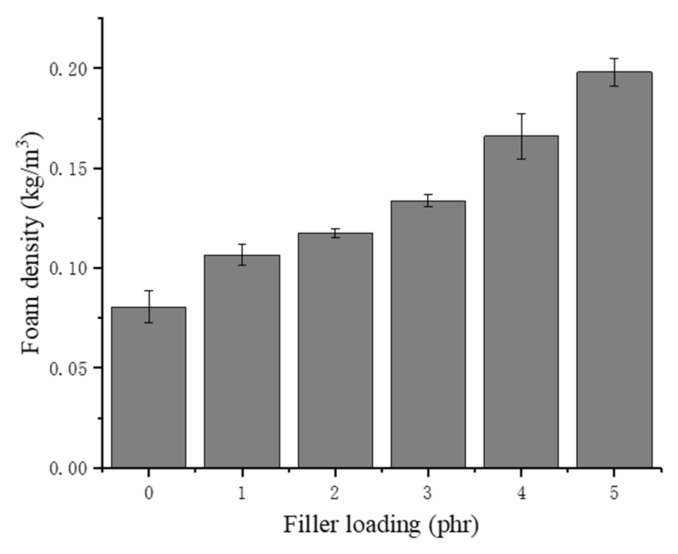
Density of chitin–NRLF composites.

As shown in Figure 5, the density of the NRLF increases with increasing chitin loading. The density of chitin particles is 1370 kg/m^3^, which is much higher than that of the pure NRLF. In addition, agglomerates destroy the original bubble structure in the NRLF and cause collapse of the cells. A decrease in bubble volume leads to a decreased proportion of air, so the NRLFs with a high loading contain additional rubber and chitin. Although the density of the NRLF composite increases as the filler content increases, it is still lower than that of the other materials. This is attributed to the unique porous structure of the NRLF. The density of the NRLF composite is still less than 0.2 kg/m^3^.

### 3.4. Chemical Composition

**Figure 6:**
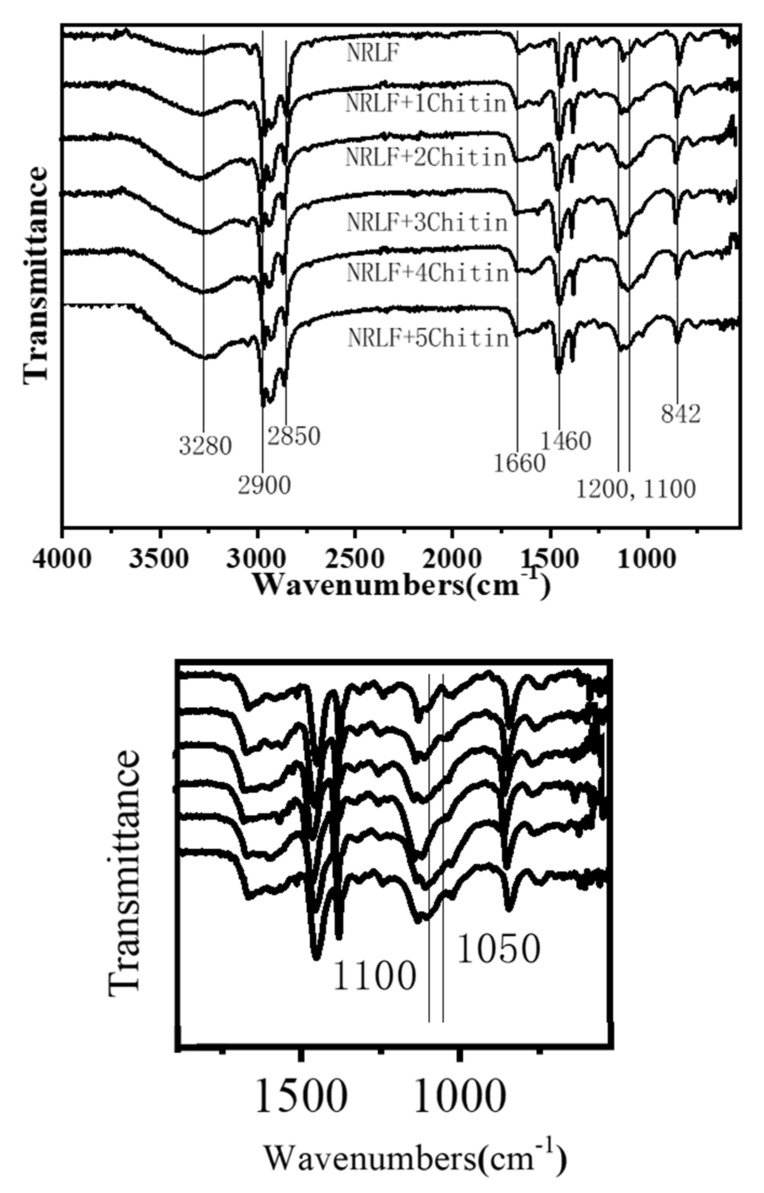
FTIR spectra of chitin–NRLF.

The FTIR transmittance spectra of the NRLFs are shown in Figure 6. FTIR spectra are used to analyze the chemical compositions and to identify the structural transformation that occurs during blending. The peak at 2900 cm^−1^ is due to the stretching vibration of the C-H bonds in methyl, the peak at 1660 cm^−1^ is due to the stretching vibration of C = C bonds, the peak at 2850 cm^−1^ is due to the stretching vibration of C-H bonds in methylene, the peak at 1460 cm^−1^ is due to the bending vibration of C-H bonds in methylene, and the peak at 842 cm^−1^ is due to the out-of-plane bending of C-H bonds [43,44,45]. These peaks are the key features in the spectra for the rubber hydrocarbons. Peaks at 3280 cm^−1^ are probably connected to moisture in the samples.

Natural rubber latex originally contains proteins, carbohydrates, lipids and other substances, hence pure NRL possesses hydroxyl and amide peaks as well. From 1200 cm^−1^ to 1000 cm^−1^, there are ether bond peaks [46,47]. This characteristic peak differentiates the chitin from the polyisoprene. As shown in the second local graph, the transmittance peaks of ether bonds are enhanced compared with pure NRLF. This demonstrates that chitin blends with the NRLF and does not significantly change the chemical structure of the natural rubber. It can be seen that with an increase in the chitin content, the natural rubber peaks in the transmittance spectra do not change. This indicates that there is no reaction between the chitin and natural rubber latex during blending and only physical loading occurs. In other words, the chitin has no significant impact on the spatial structure of the natural rubber.

### 3.5. Hardness

**Figure 7:**
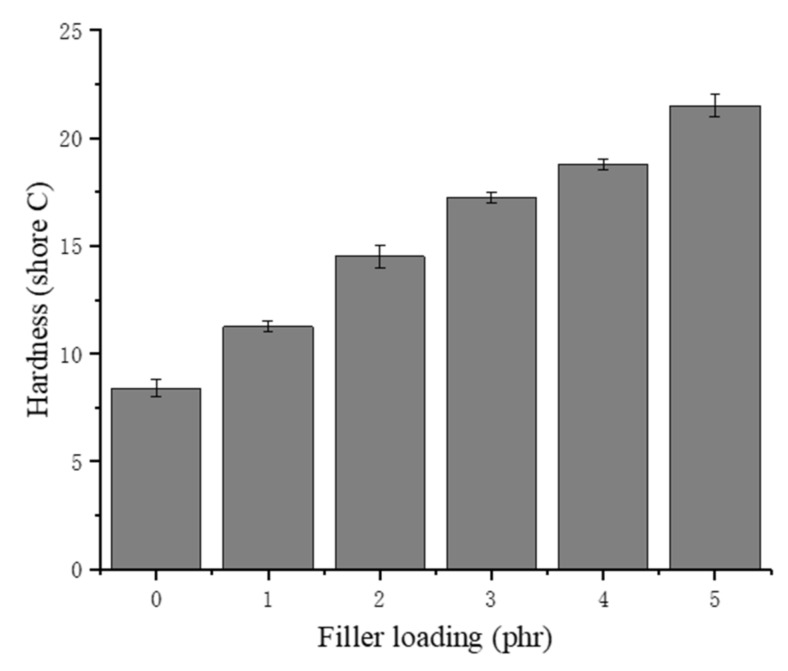
Hardness of chitin–NRLF composites.

The effects of the chitin on the hardness are presented in Figure 7. The hardness (Shore C) mainly represents the ability of rubber to resist the pressing or intrusion of hard objects. The NRLF samples with an increased chitin loading have an increased hardness, according to the trend in Figure 7. The increase in chitin content leads to the collapse and partial adhesion of the NR, so the rubber clots provide additional resistance to the external intrusion. Chitin is a linear polymer composed of (1–4) linked 2-acetamido-2-deoxy-D-glucosamine. The hardness of chitin is 7–7.5 (Mohs) due to its regularly arranged structure. The Mohs hardness is used to describe the hardness of minerals, which is absolutely higher than that of rubber. Therefore, chitin could obviously enhance the hardness of NRLF composites.

### 3.6. Tensile Strength and Elongation at Break

**Figure 8:**
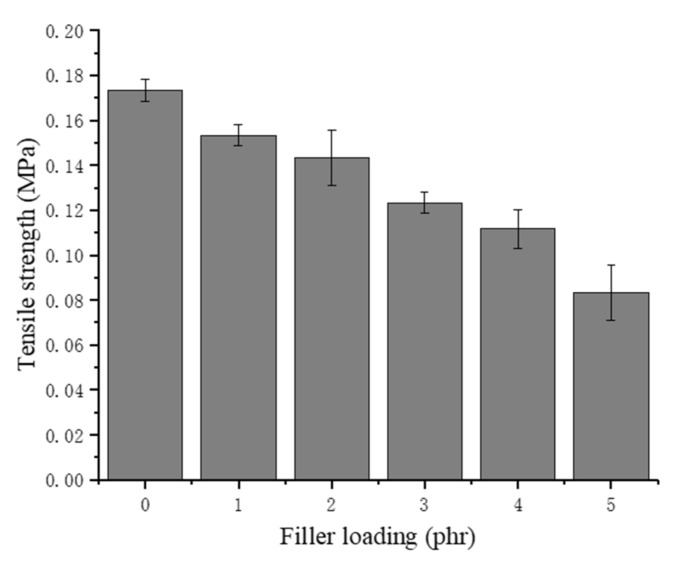
Tensile strength of chitin–NRLF.

As shown in Figure 8, the tensile strength of the chitin–NRLF composite decreases with increasing chitin content. For NRLF–5 phr chitin, the tensile strength decreases to approximately half that of the pure NRLF. This decline proves that the structure of the NRLF is destroyed during the foaming process and that additional cells appear to burst before solidification. The low compatibility between the chitin and NR leads to a large surface tension on the foam, resulting in an easy bursting of the bubbles. With increasing pore size, the cells gradually become more fragile. The increase in the filler loading could cause poor dispersion and agglomeration of the chitin. The precipitation of the chitin on the surface would form stress concentrations during tensile testing.

**Figure 9:**
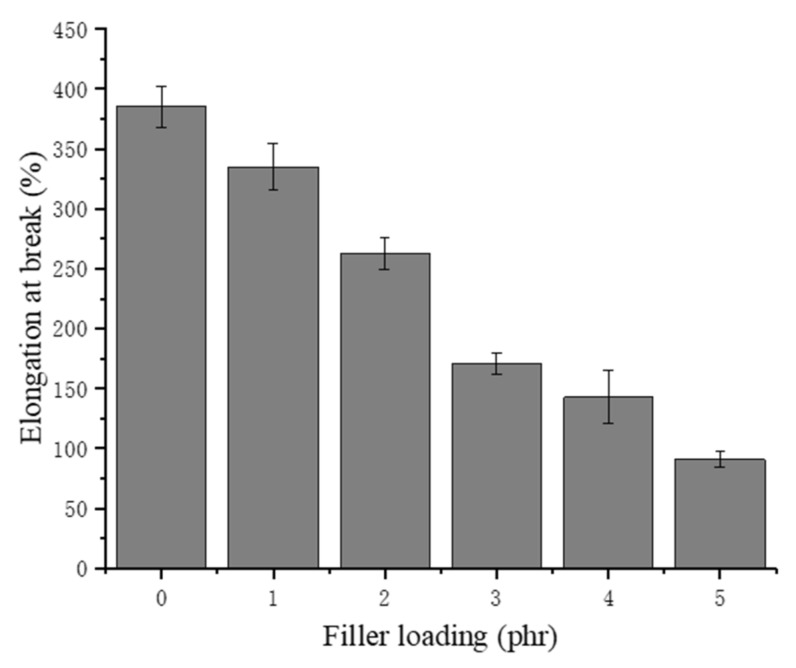
Elongation at break of chitin–NRLF.

The elongation at break of NRLFs normally depends on the crosslinking density and the foam structure. Given that the NRLF composites herein were vulcanized under the same conditions [48,49,50], the difference in elongation at break would mostly be attributed to the size and quantity of the pores. As shown in Figure 9, the elongation at break of the NRLF decreases with increasing chitin loading. Similar to the trend for the tensile strength, the elongation at break of NRLF decreases remarkably when the chitin reaches 3 phr or more. The trend of these results is analogous to other literature [30]. Stress concentrations would suddenly develop at the agglomerations of the chitin, and the thinned foam walls could accelerate the breaking. Moreover, the increased hardness of the NRLF composite might contribute to the brittleness that appears during the tensile testing, which means a decreased amount of deformation before a break occurs.

### 3.7. Compression Strength

**Figure 10:**
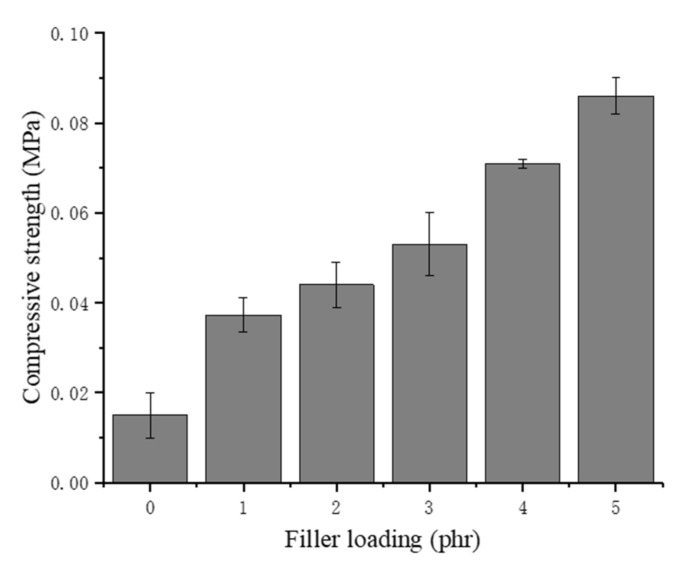
Compression strength of chitin–NRLF.

As shown in Figure 10, the compressive strength of the NRLF increases substantially with increasing chitin loading. Chitin has a much higher compression strength than natural rubber, and a small amount of chitin could dramatically enhance the compressive strength of NRLF composites. In addition, when the chitin content continues to increase, the fracture of the cell might lead to adhesion of the natural rubber. For the same volume, the NRLFs with a high loading allocate an increased proportion to rubber but a decreased proportion to air. During compression, NRLFs with a high loading would have a decreased amount of inner space available to shrink. Therefore, when the samples are compressed at 50% during the compression testing, the additional rubber and chitin particles in the NRLFs with a high loading provide an increased resistance to the pressure and increased compressive strength as well. 

### 3.8. Antibacterial Activity

**Figure 11:**
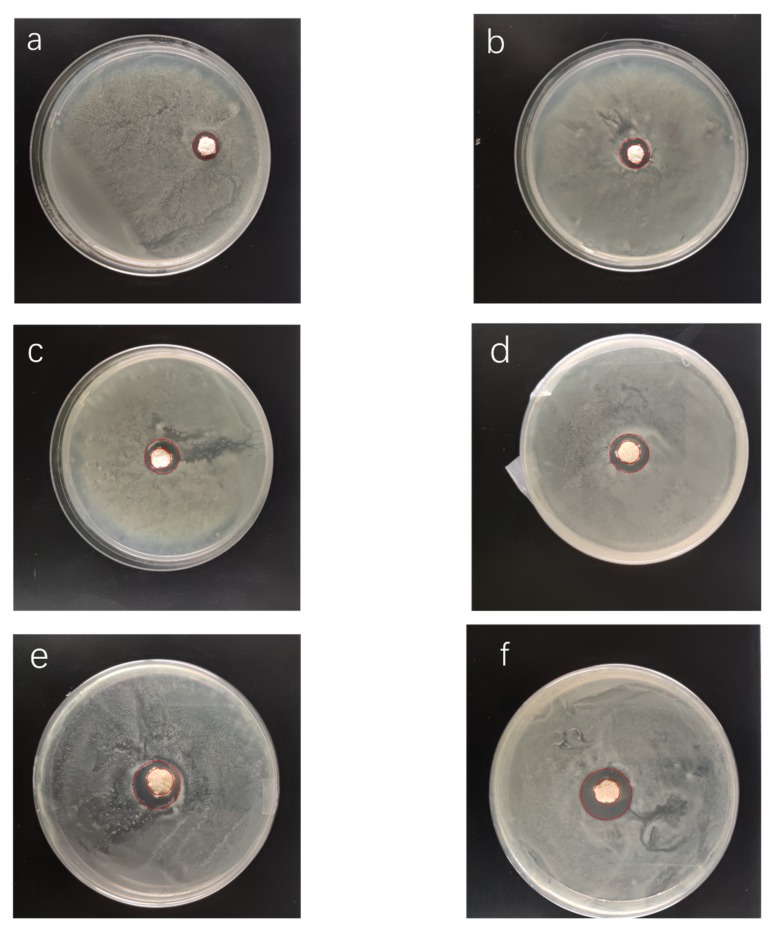
Inhibition zone against *E. coli* of chitin–NRLF composites. (**a**) pure NRLF; (**b**) NRLF–1 phr chitin; (**c**) NRLF–2 phr chitin; (**d**) NRLF–3 phr chitin; (**e**) NRLF–4 phr chitin; (**f**) NRLF–5 phr chitin.

As shown in Figure 11, the antibacterial activity of the NRLFs on *E. coli* was characterized by inhibition zone testing against *E. coli*. Figure 11a–f represents the NRLF composites filled with chitin from 0 to 5 phr.

From Figure 11a, pure NRLF without chitin already has antibacterial activity against *E. coli*. This result is consistent with the previous study [40]. There are two possible explanations for this phenomenon. First, the proteins in natural rubber latex might be bacteriostatic [51]. Second, 3 phr of zinc oxide was added, as listed in Table 1, and zinc oxide is a proven antibacterial [52,53]. 

**Figure 12:**
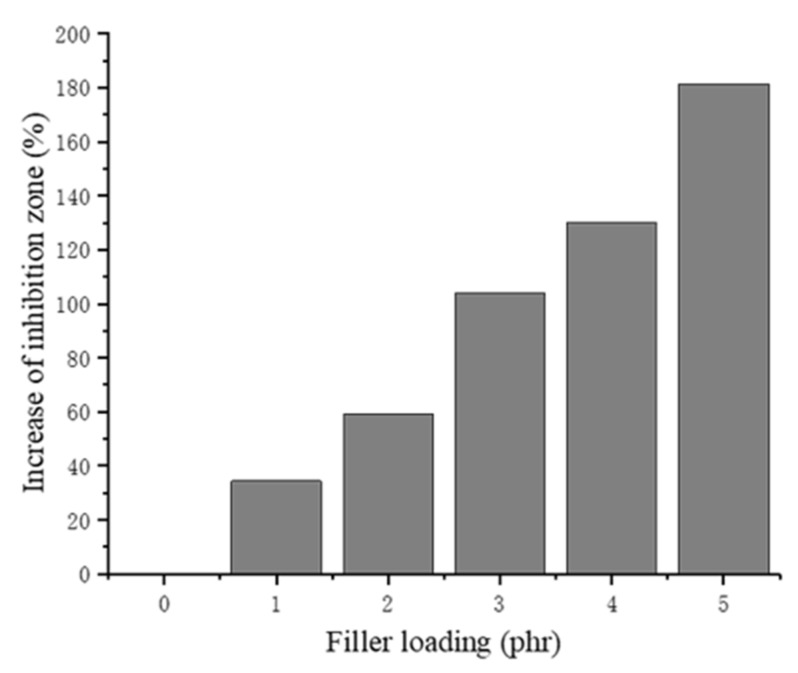
Increase in the inhibition zone of chitin–NRLF.

The increase in the inhibition zone of each of the NRLF composites can be seen in Figure 12. When the chitin loading increases, the antibacterial activity of the NRLF gradually increases. The samples were tightly embedded in the solid medium so they contacted the *E. coli* completely. The antibacterial activity of NRLF–3 phr chitin appears to be twice that of pure NRLF. When loaded with 5 phr, the inhibition zone is obviously enlarged and is quite clear compared with that of the former samples. Furthermore, the NRLF–5 phr chitin composite improved the antibacterial activity by 181.3% against *E. coli* compared to that of the pure NRLF.

## 4. Conclusions

In this research, chitin was chosen as an antibacterial agent to enhance the antibacterial activity of NRLF. NRLF composites were prepared by the Dunlop method, and chitin was blended with foam during the foaming process. In addition to the antibacterial activity, morphology, swelling, density, chemical composition, hardness, tensile strength, elongation at break and compression strength were characterized as well.

Compared with that of pure NRLF, the performance of the NRLF composite is related to the loading of chitin. When the chitin content increases, the cells expand and deform with the chitin. Overexpansion could lead to bursting of the bubbles and fracture of the rubber walls. The tensile strength, elongation at break and water swelling decrease gradually, resulting in uneven force and stress concentrations.

The chitin is inclined to aggregate due to the poor interaction between the chitin and natural rubber latex. The cracked cells and broken walls stick to the central chitin agglomerations. A decreased number of bubbles contribute to the collapse and contraction of the foam materials. With a decreased proportion of air and additional space for chitin, when the chitin loading increases, the compressive strength, density, hardness and antibacterial activity increase. 

Using the natural antibacterial agent chitin as a loading filler, environmentally friendly and antibacterial NRLFs were prepared herein. The antibacterial activity of NRLF composites could be increased by up to 181.3% compared to pure NRLF. When loaded with 3 phr chitin, the microstructure and mechanical properties of the chitin–NRLF composite change moderately and the antibacterial activity reaches more than double that of pure NRLF. Therefore, chitin–NRLF composites could be widely applied to household products, such as pillows, mattresses or cushions, and possess good practical value for future applications.

## Figures and Tables

**Table 1:** Formulation parameters for the synthesis of the chitin–natural rubber latex foam (NRLF) composites.

Ingredient	TSC	phr
natural rubber latex	0.6	100
potassium hydroxide	1	0.5
2-mercaptobenzimidazole	0.5	1.5
2-mercaptobenzothiazole	0.5	2
sulfur	0.5	2.5
potassium oleate	0.33	1
ammonium sulfate	0.4	2.5
zinc oxide	0.5	3
sodium fluorosilicone	0.5	1
chitin	1	0, 1, 2, 3, 4, 5

TSC: total solid content; phr: per hundred rubber.
